# Toward clinical translation: montmorillonite-enhanced Lactobacillus biofilm alleviates colitis by modulating the gut microbiota–bile acid axis

**DOI:** 10.1016/j.mtbio.2026.103211

**Published:** 2026-05-08

**Authors:** Zhongyuan Wang, Feng Zhu, Jie Zhang, Kangkang Feng, Waresi Abudourexiti, Song Li, Yanzhe Guo, Mingfei Chen, Zeqian Yu, Lei Zhao, Zhen Guo, Chao Ding, Jianfeng Gong

**Affiliations:** a Department of General Surgery, Jinling Hospital, Affiliated Hospital of Medical School, Nanjing University, Nanjing, China; b State Key Laboratory of Pharmaceutical Biotechnology, Chemistry and Biomedicine Innovation Center [ChemBIC], Jiangsu Key Laboratory of Molecular Medicine, Medical School of Nanjing University, Nanjing, China; c Department of Anorectal Surgery, The Quzhou Affiliated Hospital of Wenzhou Medical University, Quzhou People' s Hospital, Quzhou, Zhejiang, China; d Department of General Surgery, Jinling Clinical Medical College, Nanjing University of Chinese Medicine, Nanjing, Jiangsu, China; e Department of General Surgery, Nanjing Women and Children's Healthcare Hospital, Women's Hospital of Nanjing Medical University, Nanjing, China; f Department of General Surgery, Nanjing Drum Tower Hospital, Affiliated Hospital of Medical School, Nanjing University, Nanjing, China

**Keywords:** Ulcerative colitis, Oral probiotic delivery, Biofilm, Bile acid metabolism

## Abstract

Oral probiotics hold therapeutic potential for ulcerative colitis (UC), but their low bioavailability greatly limits clinical efficacy. Here, we designed a montmorillonite–*Lactobacillus acidophilus* biofilm (MLB) delivery strategy to enhance probiotic stability, intestinal adhesion, and therapeutic efficiency. MLB was prepared by inducing the clinically common strain *Lactobacillus acidophilus* to form biofilms on montmorillonite, an antidiarrheal agent widely used in clinics. The in vitro assays demonstrated that montmorillonite significantly promoted biofilm formation, thereby improving bacterial survival in gastrointestinal conditions and enhancing mucosal adhesion. In vivo, MLB showed superior efficacy in alleviating DSS-induced colitis compared with free bacteria or non-biofilm mixtures. Mechanistically, MLB remodeled gut microbiota composition and restored microbial bile acid metabolism through elevated bile salt hydrolase activity. This led to increased production of secondary bile acids, which in turn promoted anti-inflammatory macrophage polarization and facilitated inflammation resolution. Together, these findings demonstrate that MLB enhances the efficacy of oral probiotics by targeting the microbiota–bile acid–immune axis, representing a safe and practical approach for UC treatment.

## Graphical abstract

Graphical abstract: **The preparation, enhanced oral delivery, and therapeutic mechanism of MLB.** (A) Genetic evidence indicates a causal relationship between reduced Lactobacillus abundance and increased risk of UC. (B) The preparation of MLB. (C) Oral delivery of the MLB and their therapeutic mechanism in treating UC.

**Figure:**
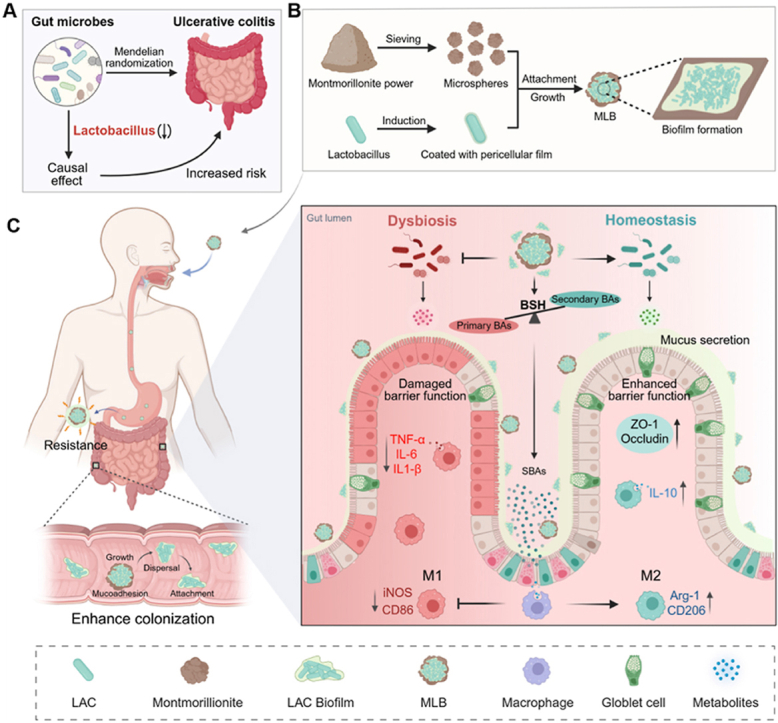

## 1 Introduction

Ulcerative colitis (UC), a predominant form of inflammatory bowel disease (IBD), manifests as recurrent inflammation limited to the mucosa and submucosa of the colon and rectum [1,2]. Despite decades of research, its pathogenesis remains incompletely understood and is thought to involve complex interactions among immune dysregulation, gut microbiota alterations, genetic susceptibility, and environmental factors [3,4]. Current therapies, including aminosalicylates, steroids, immunosuppressive drugs, and biologics, may induce remission, but their long-term efficacy is limited by high recurrence rates and considerable toxicities [[5], [6], [7]]. Driven by these limitations, there is a pressing need for UC treatment strategies that are not only safer and more efficacious but also aimed at the core pathological processes.

Gut microbiota dysbiosis has been strongly implicated in UC development [4]. This dysbiosis is hallmarked by a reduction in beneficial bacteria and an increased proliferation of potentially harmful microbes [8,9]. This microbial imbalance compromises the intestinal epithelial barrier and drives persistent mucosal inflammation [10] Accordingly, interventions targeting the gut microbiota have gained attention as a potential strategy for UC treatment [6,9]. Probiotics, primarily administered orally, are commonly applied in UC treatment due to their favorable safety profile, cost-effectiveness, and ability to regulate immune function and promote mucosal healing [11,12]. Nonetheless, the clinical efficacy of probiotics is often constrained by poor survival in the gastrointestinal tract and weak colonization, which limit their persistence and therapeutic durability in UC [[13], [14], [15]].

To overcome these limitations, delivery strategies such as microencapsulation, polymer coatings, nanoparticles, and surface modifications have been developed to improve probiotic viability in the gastrointestinal tract [13,16]. However, these approaches typically involve intricate chemical modifications that can impair probiotic activity and pose in vivo safety risks [16,17]. In addition, their complicated preparation and purification steps limit large-scale production and real-world applicability [8]. Furthermore, using a single probiotic strain as monotherapy is often insufficient to restore the disturbed gut microbiota, due to the strong colonization resistance of the native microbial community and the complex inflammatory environment present in UC [[18], [19], [20]]. These issues underscore the imperative to create novel strategies that enhance the effectiveness and practical application of oral probiotic treatments.

Recently, biofilm-based probiotic delivery strategies have garnered increasing attention [21]. Biofilms, consisting of bacterial communities encased in self-produced extracellular polymeric substances, create a protective niche that increases resilience to environmental stress [22,23]. Compared with planktonic probiotics, biofilm-forming probiotics demonstrate improved stress tolerance, mucosal adhesion, and colonization efficiency [24,25]. However, current methods for probiotic biofilm construction often lack simplicity, safety, and the use of food-grade materials [21]. In particular, the absence of natural, orally administrable carriers remains a major barrier to clinical application [21]. Recent studies have shown that montmorillonite (MMT), a natural layered silicate mineral, can interact with probiotic bacteria through surface charge-mediated mechanisms to promote extracellular polysaccharide production and facilitate the formation of structurally stable biofilms [26]. MMT is widely used in oral antidiarrheal medications and is known for its excellent gastrointestinal safety, biocompatibility, and mucoadhesive properties [26]. These characteristics make it a clinically viable and scalable platform for developing probiotic biofilm delivery systems.

**Scheme 1:**
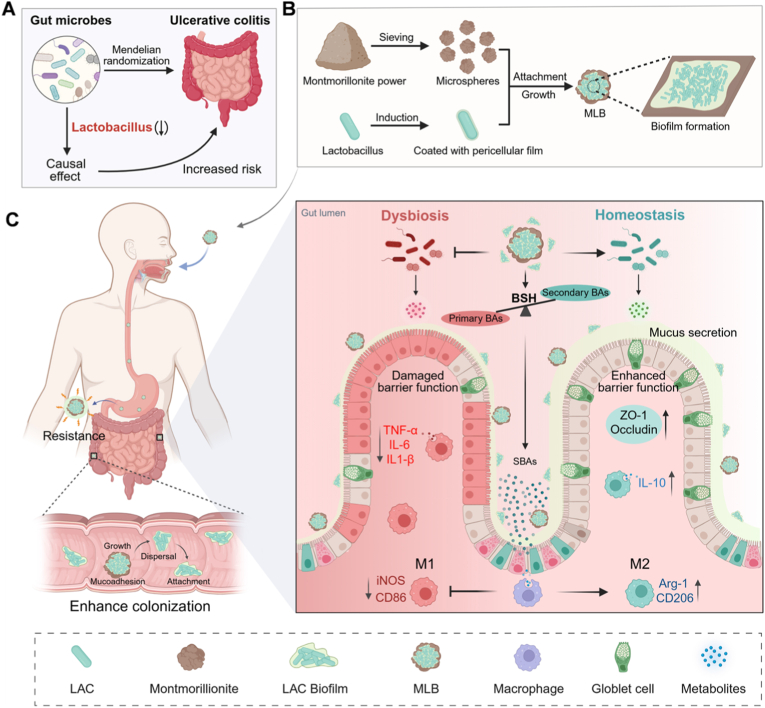
**Overview of MLB preparation, oral delivery enhancement, and therapeutic action.** (A) Genetic data link decreased Lactobacillus abundance with increased UC risk. (B) The preparation of MLB. (C) Oral delivery of MLB and its mode of action in treating UC.

Based on the above, we hypothesized that a biofilm delivery system co-constructed with MMT and a protective probiotic could serve as an effective and clinically translatable strategy for UC. Through mining publicly available datasets, we first identified *Lactobacillus* as a protective genus against UC (Scheme 1A). Accordingly, we selected the clinically approved strain *Lactobacillus acidophilus* (LAC) and co-cultured it with MMT to construct a montmorillonite–*L. acidophilus* biofilm (MLB) (Scheme 1B). This study aimed to evaluate the stability, adhesion, and therapeutic efficacy of MLB in a murine colitis model, and to explore its underlying mechanisms (Scheme 1C). Overall, this work presents a novel, safe, and convenient biofilm-based probiotic delivery system with strong potential for clinical translation.

## 2 Materials and methods

### 2.1 Mendelian randomization (MR) analysis

To explore the causal relationship with UC, we employed genetic instruments derived from genome-wide association study (GWAS) summary statistics of the gut microbiota. Variant data were obtained from the MiBioGen consortium, which includes 18,040 individuals [27]. Summary statistics for UC were derived from two sources: the GWAS dataset reported by de Lange et al., which included 12,366 cases and 33,609 controls, and the FinnGen consortium, comprising 4320 cases and 210,300 controls [28,29]. To assess causality between gut microbiota and UC, we applied multiple MR approaches, including the radial inverse-variance weighted method, weighted median, Mendelian randomization–robust adjusted profile score, and the MR pleiotropy residual sum and outlier (MR-PRESSO) test [[30], [31], [32], [33]]. Among these, radial IVW served as the primary analytical method due to its efficiency under valid MR assumptions. Results derived from the two datasets were subsequently integrated through meta-analysis. All analyses were conducted in R, using publicly available and ethically approved datasets.

### 2.2 Bacteria strain and culture

*Lactobacillus acidophilus* (ATCC 4356) was obtained from the China General Microbiological Culture Collection Center (CGMCC) and maintained either on MRS agar plates or in MRS broth under shaking conditions at 37 °C. Bacterial growth was monitored spectrophotometrically at 600 nm. To determine colony-forming units (CFUs), serial dilutions of bacterial suspensions were plated on MRS agar and incubated for 24 h at 37 °C prior to enumeration.

### 2.3 Synthesis and characterization of MLB

MMT (10–30 μm) was fractionated using 400- and 1000-mesh sieves, sterilized via autoclaving (121 °C, 20 min), and desiccated for storage. LAC was pre-cultured in MRS medium (100 mL, 37 °C, overnight) to ensure cellular activity. The activated LAC was then co-incubated with MMT for 48 h to promote biofilm formation. The MLB composite was harvested by centrifugation (4000 rpm, 5 min), then washed three times in phosphate-buffered saline (PBS) to clear residual medium.

For scanning electron microscopy (SEM) imaging, MLB was lyophilized (48 h), mounted on stubs, sputter-coated with gold, and examined under high vacuum. For biofilm fluorescence analysis, MLB samples were affixed to glass slides, dried (37 °C), and fixed in 2.5% glutaraldehyde (1 h). Staining was performed sequentially with concanavalin A (ConA) tetramethylrhodame conjugate (1 h) and DAPI (20 min). Fluorescence imaging was conducted using a Leica 980 confocal microscope.

### 2.4 Resistance of MLB against environmental assaults

To assess stress tolerance, equal amounts of LAC and MLB were resuspended in 1 mL of media containing bile salts, strong acidic solution, or subjected to heat stress while gently shaken. Digestive stress was mimicked by incubating the suspensions in pepsin solution (10 g/L in 0.85% NaCl) or in trypsin solution (10 g/L in KH_2_PO_4_ buffer) at 37 °C with constant mild shaking. Samples (100 μL) were taken at specific intervals, rinsed in PBS, and spread on MRS agar plates. CFUs were counted after 24 h incubation at 37 °C to evaluate bacterial survival.

### 2.5 Animal

Female C57BL/6J mice (6–8 weeks old) were purchased from the Laboratory Animal Center of Nanjing University. All experimental procedures were performed following institutional regulations and approved by the Institutional Animal Care and Use Committee (IACUC) of Nanjing University (Approval No. IACUC-2502001).

### 2.6 Patient samples and ethics

Intestinal specimens from six UC patients were collected during surgical resection at *Jinling* Hospital, affiliated with the Medical School of Nanjing University. Informed written consent was obtained before sampling. Ethical approval was granted by the Ethics Committee of *Jinling* Hospital, and all procedures followed the Declaration of Helsinki guidelines.

### 2.7 Evaluation of mucoadhesion

To investigate intestinal mucosal adhesion, fluorescently labeled LAC and MLB were incubated with ex vivo colonic tissues from mice and UC patients. Bacterial adherence to the mucosal surface was assessed after incubation using an in vivo imaging system (IVIS) and 3D confocal laser scanning microscopy (CLSM). Fluorescence imaging was also applied to assess in vivo retention and mucus adherence. In the animal study, female C57BL/6 mice were randomly divided into three groups (n = 3 per group) and orally administered ICG-NHS-labeled LAC, a physical mixture of LAC and MMT (ML), or MLB, each containing 1 × 10^8^ CFUs of LAC. Fluorescent signals were monitored at specified intervals using the IVIS Spectrum system. After imaging, the mice were humanely sacrificed in accordance with institutional guidelines, and colonic tissues were retrieved for subsequent experimental evaluation. Separately, another cohort of mice received RB-NHS-labeled LAC, ML, or MLB (1 × 10^8^ CFUs). At predetermined time points post-administration, colonic tissues were harvested, embedded in cryoprotective medium, and frozen-sectioned. Sections were stained with MUC-2 to visualize mucus, and the accumulation of LAC within the mucus layer was examined by CLSM fluorescence imaging.

### 2.8 Modeling and treatment of mice with colitis

Female C57BL/6 mice (6–8 weeks of age) were maintained in groups of five per cage under standard conditions, with a one-week acclimation period before experimental use. Mice were treated with 3% DSS (36,000–50,000 Da; MP Biomedicals) in drinking water for 5 days and then maintained on normal water until day 10, when they were sacrificed. Throughout the study, control animals had access solely to normal drinking water. Parallel cohorts were subjected to oral administration of PBS, MMT, LAC, ML, or MLB following the experimental schedule.

### 2.9 Assessment of disease severity

During the study, body weight was recorded daily. Disease severity was quantified using a disease activity index (DAI), calculated by scoring weight loss, stool consistency, and fecal blood (0–4 each). Upon completion of the experiment, colons were excised and their lengths measured.

### 2.10 Histological analysis

Following sacrifice, colons were removed and washed with PBS. After collection, tissues were placed in 4% paraformaldehyde at ambient temperature for 24 h and then processed for paraffin inclusion. Sections 5 μm thick were cut from paraffin-embedded tissues and stained with hematoxylin and eosin (H&E). Colonic histological damage was assessed blindly using established scoring systems. For mucus analysis, colons were triple-washed with saline and immersed in Carnoy's fixative at 4 °C overnight before processing for embedding. Alcian blue staining was performed, and images were obtained accordingly.

For immunofluorescence, tissue sections were double-labeled with antibodies against F4/80 and CD206 to detect M2 macrophages, and with F4/80 and CD86 to identify M1 macrophages. In addition, sections were immunostained for TNF-α, IL-1β, and IL-6, incubated with secondary antibodies at room temperature for 1 h, and visualized using DAB.

## 3 ELISA

Tissue homogenates of mouse colons were prepared in cold PBS with enzymes, and inflammatory cytokines were assessed using ELISA kits according to the supplied instructions.

### 3.1 Quantitative real-time PCR

Bacterial RNA was purified with a rapid extraction kit (Sangon Biotech). Tissue and cellular RNA was isolated via Trizol homogenization (Invitrogen). Quantitative real-time PCR was conducted on a 7300 system using SYBR Green I (Roche), with primers synthesized by Sangon Biotech (sequences in Table S1).

### 3.2 Biosafety assessment

In the safety study, female C57BL/6 mice (6–8 weeks old) received oral doses of MLB containing 1 × 10^8^ CFUs of LAC every other day for 10 days, with PBS-treated mice serving as controls. Body weight was tracked daily. Blood samples were obtained on day 10 to assess liver and kidney function. After euthanasia, colons were excised for length measurement, and major organs were harvested for H&E histological examination.

### 3.3 Microbiome analysis

Fecal DNA was isolated using PowerSoil® (Qiagen), quantified with Nanodrop, and checked on 1.2% agarose gels. The V3–V4 16S rRNA region was amplified with barcoded primers 341F/806 R and Pfu polymerase (Thermo Fisher). Amplicons were purified, quantified, and used to construct sequencing libraries with TruSeq Nano DNA LT Kit (Illumina). Libraries were quality-checked and sequenced on MiSeq (2 × 300 bp) or NovaSeq 6000 (2 × 250 bp). Sequences were processed for quality, denoised, merged, and chimera-filtered using QIIME2 (DADA2) or VSEARCH. OTUs were clustered at 97% similarity and taxonomically assigned using SILVA/Greengenes. Alpha and beta diversity analyses (Shannon, Simpson, PCA) were conducted, microbial composition visualized with heatmaps, and functional prediction performed using PICRUSt2.

### 3.4 Fecal microbiota transplantation

To induce gut microbiota depletion, mice received metronidazole, neomycin, vancomycin, and ampicillin (1, 1, 0.5, and 1 g/L, respectively) in drinking water for seven days, followed by 3% DSS treatment. For FMT treatment, fecal samples were transferred into an anaerobic chamber and homogenized in pre-reduced sterile Ringer working buffer to a final concentration of 100 mg/mL. The homogenate was then passed through a 100 μm filter, and the resulting supernatant was mixed with an equal volume of 20% (w/v) skimmed milk as a cryoprotectant. The freshly prepared inoculum was used for the initial transplantation, while the remaining portion was stored at −80 °C for subsequent administrations. Following antibiotic pretreatment, mice were orally gavaged with 200 μL of the fecal suspension once daily for seven consecutive days.

### 3.5 Bile acid detection

Bile acids in fecal samples were analyzed by Suzhou PANOMIX Biomedical Tech Co., Ltd. Briefly, samples were mixed with pre-chilled methanol and glass beads, homogenized, sonicated, and centrifuged. The supernatant was diluted, filtered, and transferred to vials for UPLC–MS analysis using an ACQUITY BEH C18 column under a water–acetonitrile gradient with 0.1% formic acid. Compounds were detected in negative electrospray mode with MRM, quantified via calibration curves, and data processed using Analyst software.

### 3.6 Determination of BSH activity

BSH activity was quantified using a commercially available ELISA kit (G0933W, Grace Biotechnology) in strict accordance with the manufacturer's protocol.

### 3.7 BSH inhibitor and bile acids treatment

For BSH inhibitor treatment, GR-7 (HY-135747, MedChemExpress), a bile salt hydrolase (BSH) inhibitor, was prepared in corn oil with 5% dimethyl sulfoxide. At scheduled time points, mice were administered 200 μL of GR-7 solution (10 mg/kg) via oral gavage, whereas control animals received the vehicle only. For bile acid treatment, mice received a mixture of secondary bile acids (SBAs), with each individual acid administered at a dose of 10 mg/kg/day [34].

### 3.8 Macrophage polarization

RAW 264.7 macrophages were grown in DMEM containing 10% FBS and 1% penicillin-streptomycin at 37 °C with 5% CO_2_. For experiments on macrophage polarization, cells were pretreated with 20 μM SBAs before 24-h stimulation with LPS (500 ng/mL).

### 3.9 Cell immunofluorescence

RAW 264.7 cells were plated on 24-well plates containing 10 mm coverslips and treated as indicated. Post-treatment, cells were washed with PBS, fixed in 4% paraformaldehyde for 30 min, and blocked with 5% BSA for 30 min at room temperature. Primary antibodies against Arginase-1 and iNOS were incubated overnight at 4 °C, followed by secondary antibody staining (Alexa Fluor® 488 goat anti-rat IgG and Alexa Fluor® 546 donkey anti-rabbit IgG, 1:200) for 1 h. Nuclei were labeled with DAPI for 10 min, and immunofluorescence images were acquired using a Zeiss LSM980 microscope.

### 3.10 Western blot analysis

RAW 264.7 cells were seeded in 6-well plates and treated as indicated. After 24 h, total protein was extracted using a Beyotime kit and quantified with BCA assay. Samples were combined with 5 × SDS loading buffer, boiled at 100 °C for 10 min, and separated on 12% SDS-PAGE gels. Proteins were transferred to PVDF membranes at 300 mA for 2 h, blocked with 5% BSA for 1 h, and incubated overnight at 4 °C with primary antibodies. Following TBST washes, HRP-conjugated secondary antibodies were applied for 1 h, and protein signals were detected using a Tanon-5200 chemiluminescent imaging system.

### 3.11 Statistical analysis

Data are presented as mean ± SEM. Comparisons between two groups were performed using two-tailed Student's t-test, while differences among multiple groups were analyzed by one-way ANOVA. Statistical significance was defined as *p* < 0.05 (*p*< 0.05: ∗, *p*< 0.01: ∗∗, *p*< 0.001: ∗∗∗, *p*< 0.0001: ∗∗∗∗).

## 4 Results

### 4.1 Causal link between *lactobacillus* abundance and UC susceptibility

**Fig. 1:**
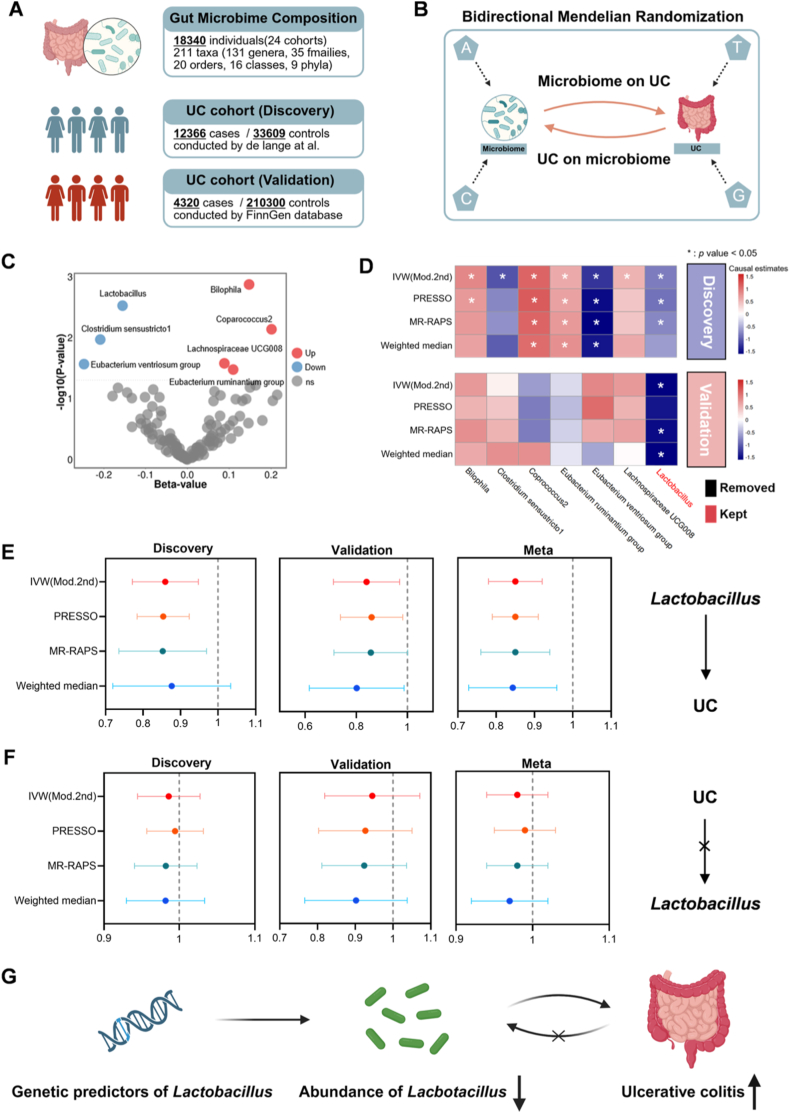
**Mendelian randomization demonstrates that Lactobacillus abundance is causally related to UC risk.** (A) Publicly available GWAS datasets on gut microbiota and UC were utilized for analysis. (B) Schematic overview of the bidirectional two-sample MR approach utilized to examine causal effects. (C) Causal estimates (β) from taxa showing significant associations in the discovery cohort using radial inverse variance weighting (*P*< 0.05). (D) Microbial taxa with significant effects in the discovery phase were further validated in an independent cohort. Asterisks (∗) denote MR significance. Positive and negative causal directions are indicated by red and blue color gradients, respectively. Taxa shown in black font were excluded based on validation results, while red font highlights those with consistent causal associations. (E, F) Odds ratios of the Lactobacillus–UC association across various MR methods in the discovery and validation cohorts, as well as in the combined meta-analysis. (G) Conceptual diagram illustrating the inferred causal pathway: genetically predicted reduction in Lactobacillus abundance contributes to elevated UC risk. (For interpretation of the references to color in this figure legend, the reader is referred to the Web version of this article.)

A summary of the study design is shown in Fig. 1A. Genetic variants identified from GWAS summary data served as instruments for gut microbial taxa, which were then analyzed using two-sample MR to evaluate their causal impact on UC risk (Fig. 1B) [35]. We initially employed the radial inverse-variance weighted method, selected for its superior statistical efficiency, to identify taxa potentially causally linked to UC using data from the GWAS by de Lange et al. [36,37] The analysis showed that higher levels of *Lactobacillus, Clostridium sensu stricto 1*, and the *Eubacterium ventriosum group* correlated with reduced UC risk, whereas elevated *Bilophila*, *Coprococcus 2, Lachnospiraceae UCG008,* and the *Eubacterium ruminantium group* were linked to increased risk (Fig. 1C and D).

To validate these findings, we conducted a replication analysis using an independent Finnish UC cohort, which confirmed the significant inverse association between *Lactobacillus* abundance and UC risk. However, associations involving the other taxa failed to achieve statistical significance in the replication dataset (Fig. 1D and E). To address potential reverse causation, we conducted a reverse MR analysis treating UC as the exposure and *Lactobacillus* abundance as the outcome, which showed no causal effect of UC on *Lactobacillus* levels (Fig. 1F). These results were reinforced through various MR methods and meta-analysis, confirming their reliability (Fig. 1D–F). Overall, our study offers genetic evidence that *Lactobacillus* serves as a protective factor in UC development, highlighting its potential for microbiota-based therapies (Fig. 1G).

### 4.2 Preparation and characterization of MLB

These findings further prompted us to develop a UC therapeutic to efficiently supplement *Lactobacillus* to the intestine [38]. To accomplish this, we chose LAC as a representative strain whose role in maintaining intestinal barrier function and regulating mucosal and systemic immune responses has been demonstrated from numerous studies [18,39,40]. Notably, LAC grown in standard MRS medium failed to form biofilms [26]. To induce biofilm formation, MMT was introduced as both an adhesion substrate and an inducer of biofilm development. This process resulted in a dense LAC biofilm on MMT particles, termed MLB.

**Fig. 2:**
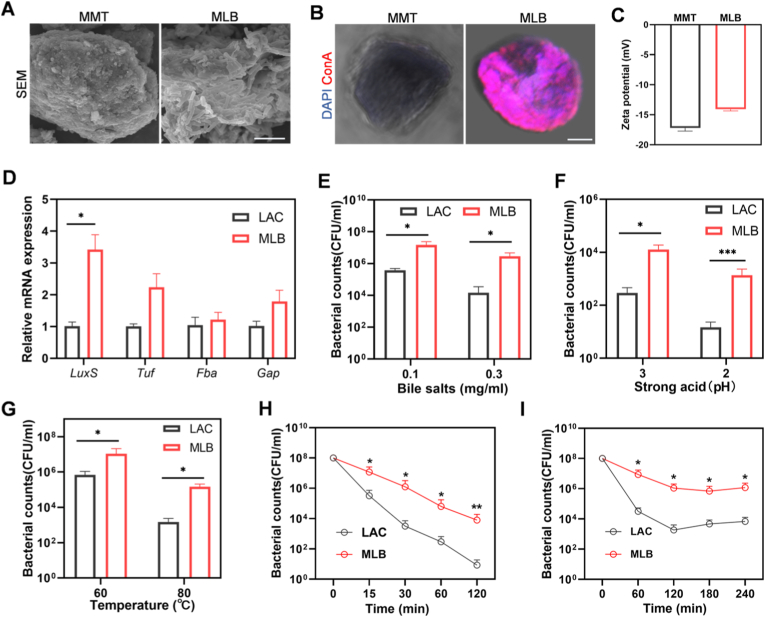
**MLB preparation, characterization, and in vitro resistance to environmental assaults.** (A) SEM images of Lactobacillus biofilms on MMT surfaces and (B) ConA staining (red) highlighting the biofilm matrix. (C) Zeta potential analysis of MMT and MLB. (D) Detection of quorum sensing genes related to biofilm. (*E*-G) In vitro bacterial resistance to (E) bile salts, (F) strong acid, (G) high temperature, (H) SGF and (I) SIF. Scale bars, 5 μm. Data are expressed as the mean ± SEM. ∗*P*< 0.05, ∗∗*P*< 0.01, ∗∗∗*P*< 0.001, ∗∗∗∗*P* < 0.0001. (For interpretation of the references to color in this figure legend, the reader is referred to the Web version of this article.)

To confirm the formation of a biofilm, we performed characterization of MLB in detail. SEM revealed no bacterial presence on the surface of MMT alone (Fig. 2A), whereas co-culture with LAC produced a distinct biofilm morphology on the MMT surface (Fig. 2B). Biofilm formation was further confirmed by the ConA staining, which showed strong signals on MLB but not on MMT (Fig. 2C). In addition, the zeta potential of MLB differed significantly from that of MMT, likely due to the presence of a surface biofilm layer (Fig. 2C). Additionally, the quorum sensing gene *LuxS*, crucial for *Lactobacillus* biofilm formation, was markedly upregulated in the presence of MMT, confirming enhanced biofilm development (Fig. 2D).

One of the hallmark features of biofilms is increased resistance to environmental stress. We therefore hypothesized that MLB would confer greater resilience to external challenges. To test this, both free LAC and MLB were exposed to acidic conditions, bile salts, and elevated temperatures. As shown in Fig. 2E–G, MLB significantly enhanced the survival of LAC under extreme pH, bile salts, and thermal stress. Building on these results, we next examined whether MLB could enhance bacterial survival in simulated gastrointestinal environments. When exposed to SGF, free LAC exhibited a time-dependent decline in viability, while MLB maintained significantly higher survival even after 2 h of exposure (Fig. 2H). Similarly, exposure to SIF for 4 h resulted in substantial loss of viability in free LAC cells, whereas MLB retained high levels of viable bacteria (Fig. 2I). These results collectively indicate that MLB confers enhanced resistance to gastrointestinal environments, supporting its potential as an effective oral delivery system for UC treatment.

### 4.3 Enhanced colonization of MLB after oral administration

**Fig. 3:**
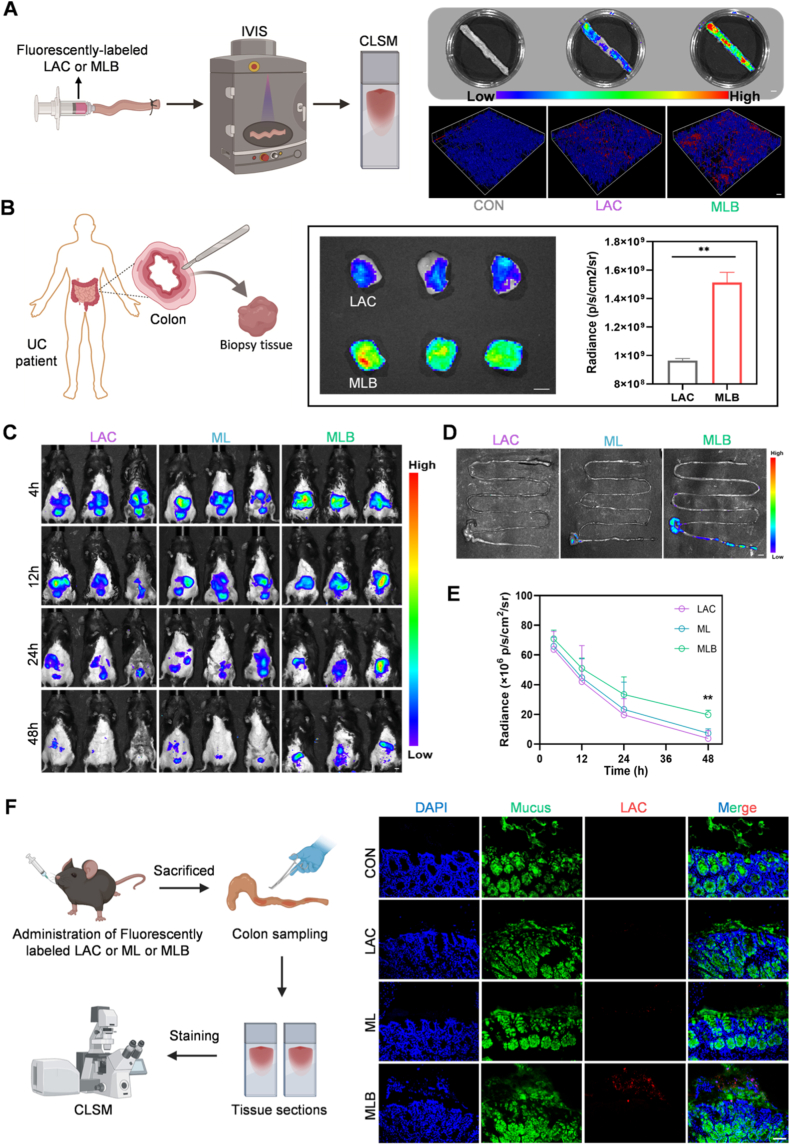
**MLB enhances probiotic mucoadhesion and colonization in the intestine.** (A) Ex vivo evaluation of adhesion to murine colonic tissue. Shown on the right are fluorescence images of full colons alongside enlarged 3D CLSM reconstructions. (B) Fluorescence images and abdominal signal quantification from ex vivo adhesion assays using UC colonic biopsies. (C) In vivo tracking of mucoadhesion following oral administration of fluorescently labeled probiotics. (D) Ex vivo imaging of intestinal distribution after treatment; representative bioluminescence images of isolated intestines corresponding to (C). (E) Quantification of abdominal fluorescence intensity from (C). (F) Fluorescence imaging of LAC retention within the mucus layer. Mucin-2 (green) marks the mucus, while rhodamine (red) labels LAC cells. The scale bars for section staining and IVIS are 50 μm and 5 mm, respectively. Data are expressed as the mean ± SEM. ∗*P*< 0.05, ∗∗*P*< 0.01, ∗∗∗*P*< 0.001, ∗∗∗∗*P* < 0.0001. (For interpretation of the references to color in this figure legend, the reader is referred to the Web version of this article.)

We evaluated the intestinal adhesion capability of MLB through in vitro experiments. Briefly, fluorescently labeled LAC and MLB were incubated with ex vivo mouse colon tissues. After incubation, bacterial adhesion to the mucosal surface was assessed using an IVIS. Fig. 3A shows that the MLB group displayed intense fluorescence, while the control groups showed considerably weaker signals. Additionally, three-dimensional reconstructed CLSM images revealed a higher number of adherent LAC cells on the intestinal wall surface in the MLB group (Fig. 3A). To evaluate MLB's mucosal adhesion, we next incubated it with colonic biopsy samples obtained from UC patients. As expected, fluorescence signals were considerably stronger in the MLB group than in the LAC group (Fig. 3B). These findings demonstrate that MLB adheres to the colonic mucosa far more effectively.

After confirming in vitro that the MLB enhances adhesion and retention, we further investigated its potential to improve probiotic colonization in vivo. To this end, mice received a single oral dose of fluorescently tagged LAC, ML, or MLB. Measurement of abdominal fluorescence showed that MLB produced markedly higher signals and retained them longer compared with the other groups (Fig. 3C–E). Although individual variations in fluorescence signals were observed within the group (potentially due to biological heterogeneity such as differences in gut microbiota composition), the mean fluorescence intensity of the MLB group remained significantly higher than that of the other groups. This prolonged retention is likely attributed to improved adhesion to the mucosal layer. At 48 h post-administration, mice were sacrificed, and intestinal fluorescence was captured with the IVIS imaging system. As shown in Fig. 3D, the MLB group exhibited markedly higher fluorescence intensity than other groups, predominantly localized in the colonic region. Given that UC-associated intestinal lesions are primarily localized in the colon, MLB is particularly well-suited for colitis-targeted delivery. Furthermore, frozen sections were used to visualize LAC adhesion in the colon, revealing extensive LAC attachment on the mucus layer in the MLB group, whereas LAC was sparsely distributed in the mucus layers of other groups (Fig. 3F). Collectively, these findings indicate that MLB markedly improves mucosal adhesion and colonization in the colon.

### 4.4 Efficacy of MLB in treating DSS-induced colitis

**Fig. 4:**
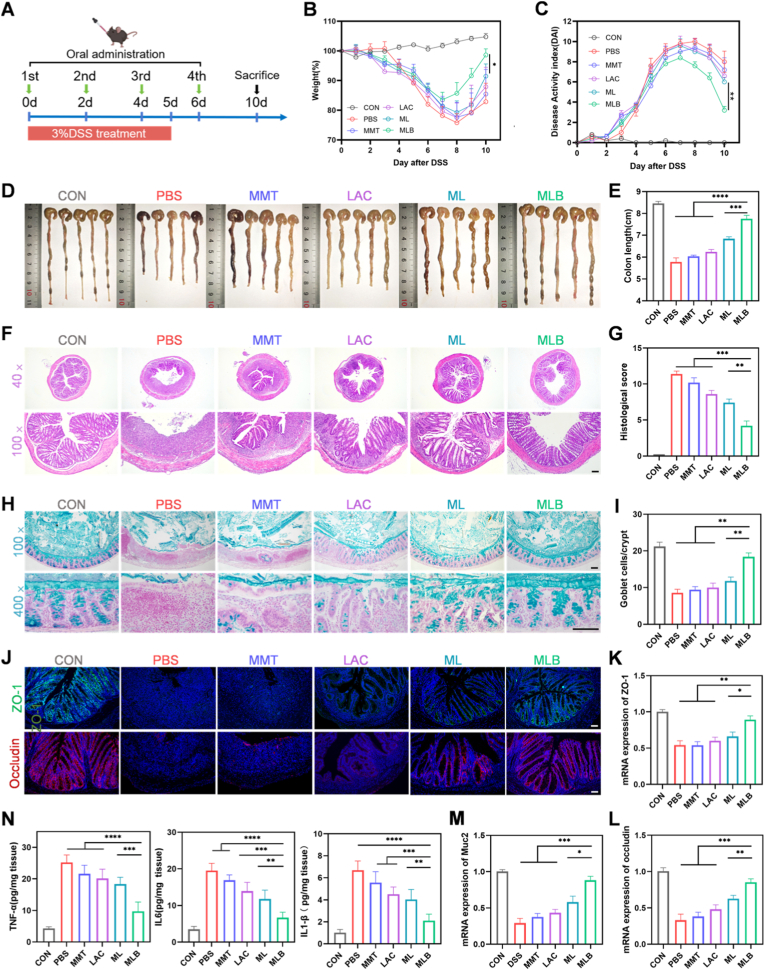
**Therapeutic efficacy of MLB in DSS-induced colitis.** (A) Experimental scheme: C57BL/6 mice received 3% DSS (day 0–5) and were gavaged with PBS, MMT, LAC, ML, or MLB (1 × 10^8 CFU) on days 0, 2, 4, and 6. (B) Body weight changes. (C) Disease activity index (DAI). (D, E) Colon morphology and length. (F, G) H&E staining and histological scores. (H, I) Alcian blue staining and goblet cell counts per crypt. (J) Immunofluorescence of tight junction proteins. (K, L) mRNA expression of tight junction proteins. (M) mRNA expression of MUC2. (N) Colonic cytokine levels (TNF-α, IL-6, IL-1β) determined by ELISA. Scale bars, 100 μm. Data are mean ± SEM (n = 5). ∗*P*< 0.05, ∗∗*P*< 0.01, ∗∗∗*P*< 0.001, ∗∗∗∗*P* < 0.0001. (For interpretation of the references to color in this figure legend, the reader is referred to the Web version of this article.)

Encouraged by the above findings, we further evaluated the therapeutic effects of MLB on DSS-induced colitis in mice. Acute colitis was induced in C57BL/6 mice by administering 3% DSS in drinking water for 5 days, followed by replacement with regular water (Fig. 4A). DSS administration (PBS + 3% DSS) induced classic IBD features in mice—such as body weight loss, higher DAI scores, shortened colons, tissue damage, and increased inflammatory cytokines—compared with controls (PBS + water), validating the DSS colitis model (Fig. 4B–N).

To assess therapeutic potential of MLB, colitis mice were orally administered PBS, MMT, LAC, ML, or MLB (1 × 10^8^ CFU/mouse) on days 0, 2, 4, and 6 (Fig. 4A; Supplementary Fig. 5A–E). Treatment effects were assessed via body weight, DAI, and colon length. Mice receiving MLB showed notably less weight loss, decreased DAI, and longer colons compared with LAC- and ML-treated groups (Fig. 4B–E). The therapeutic effects of MLB were further confirmed by histological analysis using H&E staining. Consistent with earlier findings, DSS treatment caused marked epithelial injury, crypt disruption, and heavy infiltration of inflammatory cells. In contrast, mice receiving MLB showed milder ulceration, decreased inflammatory infiltration, and largely intact crypt architecture, as indicated by histological scores and representative images (Fig. 4F and G). Inflammatory status was further evaluated by quantifying colonic levels of IL-1β, TNF-α, and IL-6 using ELISA. As shown in Fig. 4N, DSS treatment significantly upregulated these cytokines, whereas MLB administration markedly reduced their levels, highlighting its potent anti-inflammatory effects. Next, we evaluated the impact of different formulations on colonic mucosal integrity in DSS-induced colitis. Goblet cells and their mucins are vital for preserving the colonic mucus barrier. In DSS-treated mice, MLB treatment protected goblet cells and maintained a thicker mucus layer (Figure H, I). Additionally, tight junction proteins ZO-1 and occludin, key for barrier integrity, were restored by MLB, highlighting its protective effect on the colonic mucosal barrier (Fig. 4G–M).

Additionally, we conducted a series of biosafety assessments. Mice were orally administered MLB every other day, while control mice received PBS. Ten days post-treatment, blood and principal organs were obtained for further assessment. MLB-treated mice showed no significant reductions in body weight or colon length (Supplementary Fig. 1A and B). H&E staining of major organs revealed normal morphology and structure, further demonstrating the excellent biocompatibility of MLB (Supplementary Fig. 1C and D). Biochemical analysis of serum showed that liver and kidney function markers were comparable between the MLB and PBS groups, suggesting that MLB treatment did not cause hepatic or renal toxicity (Supplementary Fig. 1E and F).

### 4.5 The ability of MLB to regulate gut microbiota

These results suggest that MLB exerts its therapeutic benefits mainly by promoting the persistence of live probiotics in the gut and reshaping the intestinal microbiota. To test this hypothesis, we administered heat-inactivated MLB to DSS-induced colitis mice (Supplementary Fig. 2A). The inactivated formulation exhibited negligible therapeutic effects, further supporting the concept that the beneficial actions of MLB largely depend on the increased colonization of live probiotic bacteria (Supplementary Fig. 2B).

**Fig. 5:**
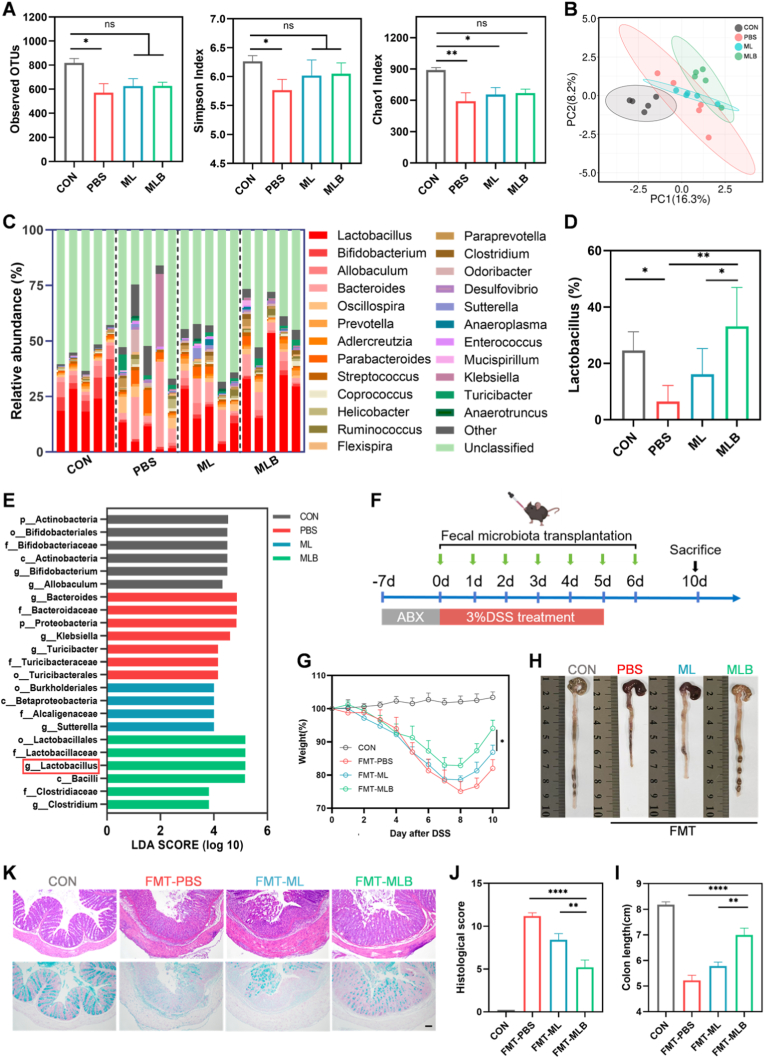
**MLB modulates gut microbiota during colitis treatment.** (A) Observed OTUs and α-diversity indices (Simpson, Chao) of gut microbiota. (B) PCA analysis of β-diversity. (C) Relative abundance of gut microbiota at genus level. (D) Relative abundance of Lactobacillus. Data are mean ± SD (n = 5). (E) LDA score plot of differentially enriched taxa (LDA >3.5). (F, G) Experimental design and body weight changes of mice following FMT. (H–K) Colon morphology, H&E and Alcian blue staining, histological scores, and colon length after FMT treatments. Scale bars, 100 μm. Data are mean ± SEM (n = 5), except for panel D. ∗*P*< 0.05, ∗∗*P*< 0.01, ∗∗∗*P*< 0.001, ∗∗∗∗*P* < 0.0001. (For interpretation of the references to color in this figure legend, the reader is referred to the Web version of this article.)

To explore how MLB modulates gut microbiota, fecal samples were harvested on day 10 for 16S rRNA sequencing. Microbial α-diversity was evaluated using observed OTUs, Shannon, and Chao1 indices. In contrast to healthy controls, PBS-treated DSS mice showed markedly decreased microbial richness and diversity (Fig. 5A). However, MLB treatment effectively restored these indices, with no significant differences compared to the healthy controls (Fig. 5A). PCA of microbial profiles demonstrated that the MLB group formed a separate cluster, reflecting a marked alteration in community structure (Fig. 5B).

We next examined the relative abundance of major bacterial phyla and genera. Consistent with previous reports in UC patients, DSS-induced colitis in mice resulted in reduced Firmicutes and elevated Proteobacteria (Supplementary Fig. 3A–C). MLB treatment restored microbial homeostasis by increasing the abundance of Firmicutes and reducing Proteobacteria (Supplementary Fig. 3A–C). At the taxonomic level of genus, analysis of the 25 most abundant genera showed that MLB treatment markedly enriched beneficial taxa, including Lactobacillus and Clostridium, while suppressing potentially pathogenic genera such as Bacteroides (Fig. 5C and D). Consistently, linear discriminant analysis (LDA) identified *Lactobacillus* as a key discriminative genus enriched in the MLB group, further validating the reshaped microbial composition observed following treatment (Fig. 5E). Notably, the abundance of *Lactobacillus* in the MLB group was significantly higher than that in the ML group, likely due to enhanced probiotic colonization protected by the biofilm matrix in MLB.

To further validate the microbiota-mediated effects, FMT was performed. After antibiotic administration, fecal samples were collected and subjected to bacterial plating assays to confirm effective depletion of the gut microbiota before transplantation. Subsequently, fecal samples collected on day 10 from each donor group were orally administered to antibiotic-pretreated colitis mice (Fig. 5F). Mice receiving fecal transplants from the MLB group showed reduced weight loss and longer colons (Fig. 5G–I). H&E histology showed reduced tissue damage in the MLB-FMT group, further supporting MLB's therapeutic efficacy (Fig. 5J and K). Notably, 16S rRNA sequencing analysis of fecal samples after FMT demonstrated a significantly increased relative abundance of *Lactobacillus* in the MLB-FMT group compared with the other groups, indicating successful microbial reconstruction and enrichment of beneficial taxa (Supplementary Fig. 6A–F). Taken together, these results demonstrate that MLB treatment significantly enhances *Lactobacillus* abundance, reshapes gut microbial composition, and contributes to the amelioration of colitis.

### 4.6 The ability of MLB to regulate bile acid metabolism

**Fig. 6:**
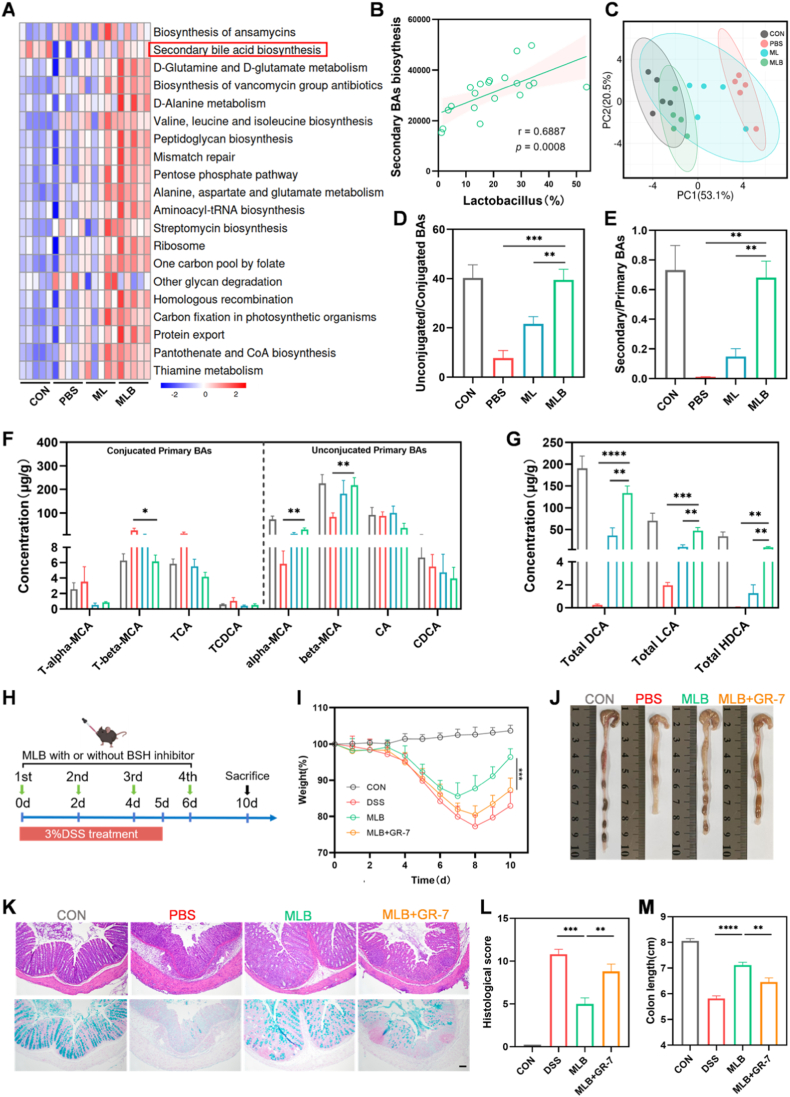
**MLB alleviates colitis by modulating bile acid metabolism. (**A) KEGG enrichment of microbial gene functions (top 20 pathways). (B) Correlation between Lactobacillus abundance and secondary bile acid biosynthesis. (C) PLS-DA of fecal bile acid profiles. (D, E) Ratios of unconjugated/conjugated and secondary/primary bile acids. (F, G) Fecal concentrations of primary bile acids and secondary bile acids (LCA, DCA, HDCA). (H) Experimental design of BSH inhibitor treatment with GR-7. (I) Body weight changes during treatment. (J–M) Colon morphology, H&E and Alcian blue staining, histological scores, and colon length after GR-7 intervention. Scale bars, 100 μm. Data are mean ± SEM (n = 5). ∗*P*< 0.05, ∗∗*P*< 0.01, ∗∗∗*P*< 0.001, ∗∗∗∗*P* < 0.0001. (For interpretation of the references to color in this figure legend, the reader is referred to the Web version of this article.)

Gut microbiota influence host physiology and pathophysiology through the production of metabolic intermediates [10]. KEGG pathway analysis revealed that MLB administration significantly altered several microbial metabolic pathways, among which bile acid metabolism emerged as one of the most affected (Fig. 6A). Recent studies have demonstrated that *Lactobacillus* species contribute to bile acid metabolism via the expression of BSH [41]. Consistently, our results showed a positive correlation between fecal *Lactobacillus* abundance and the microbial capacity for SBAs biosynthesis (Fig. 6B).

Considering the importance of bile acid metabolism in UC, we conducted targeted bile acid metabolomics to examine how MLB influences intestinal bile acid composition [42]. A heatmap of fecal bile acid abundance revealed distinct patterns across treatment groups (Supplementary Fig. 4A). PLS-DA showed distinct clustering, with the MLB group's bile acid profile approaching that of healthy controls (Fig. 6C). Additionally, colitis mice exhibited decreased total bile acid levels, which were partially restored following MLB treatment (Supplementary Fig. 4B). Notably, MLB markedly lowered conjugated bile acid levels and elevated the unconjugated-to-conjugated bile acid ratio (Fig. 6D; Supplementary Fig. 4C), indicating increased gut BSH activity. Consistently, quantitative analysis of BSH activity demonstrated that MLB significantly enhanced fecal BSH activity (Supplementary Fig. 4F). Given that BSH is predominantly expressed by *Lactobacillus*, this elevated activity is likely attributed to the increased abundance of *Lactobacillus* in the MLB group [41]. Because primary conjugated bile acids must first be deconjugated before being converted into SBAs, we next assessed primary bile acid composition [43]. In MLB-treated mice, primary conjugated bile acids were reduced, while their unconjugated forms were increased (Fig. 6F). Interestingly, levels of unconjugated primary bile acids, including cholic acid and chenodeoxycholic acid, were lower in the MLB group, likely reflecting their microbial conversion into secondary bile acids by taxa like *Clostridium*, which were enriched following MLB treatment (Figs. 5E and 6F) [44]. Consistent with these findings, MLB treatment markedly elevated SBAs—including deoxycholic acid (DCA), lithocholic acid (LCA), and hyodeoxycholic acid (HDCA)—and raised the secondary-to-primary bile acid ratio, closely resembling the profile observed in healthy controls (Fig. 6E–G; Supplementary Fig. 4D and E).

To investigate whether the production of SBAs was BSH-dependent, we performed BSH inhibition experiments by co-administering the BSH inhibitor GR-7 with MLB (Fig. 6H) [45]. GR-7 treatment substantially diminished MLB's therapeutic benefits, leading to greater weight loss, shorter colons, and more severe histological damage compared with MLB alone (Fig. 6I–M). Furthermore, supplementation with SBAs effectively alleviated colitis severity and restored a substantial proportion of the therapeutic effects observed with MLB treatment (Supplementary Fig. 7A–E). ​These findings suggest that MLB promotes BSH activity, enhancing the generation of anti-inflammatory SBAs that help alleviate colonic inflammation.

### 4.7 MLB regulates macrophage polarization to relieve colitis

The pathological microenvironment of UC is characterized by disrupted immune homeostasis, prominently featuring infiltration of pro-inflammatory immune cells [46]. Among them, macrophages accumulate in the intestinal mucosa and predominantly polarize toward the M1 phenotype, producing pro-inflammatory cytokines that exacerbate the intestinal inflammatory milieu [47,48]. In this study, MLB treatment markedly lowered levels of key pro-inflammatory cytokines—IL-6, TNF-α, and IL-1β—mainly produced by macrophages (Fig. 4N). This prompted us to propose that MLB alleviates intestinal inflammation by influencing macrophage polarization in the gut.

**Fig. 7:**
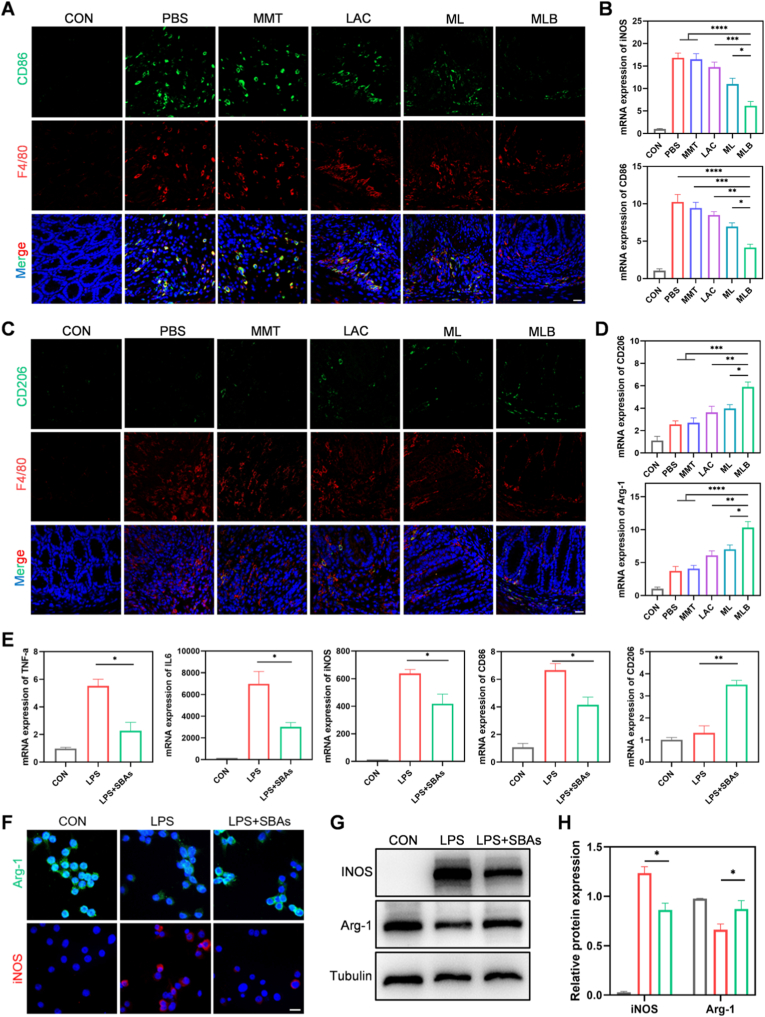
**MLB alleviates colitis by modulating macrophage polarization.** (A, C) Representative colon immunofluorescence for F4/80 (macrophages), CD86 (M1), and CD206 (M2). (B, D) Colon mRNA levels of M1 (CD86, iNOS) and M2 (CD206, Arg-1) markers across groups. (E) Corresponding mRNA in treated RAW264.7 cells. SBAs: LCA, DCA, HDCA, and 12-ketoLCA mix. (F) Macrophage iNOS/Arg-1 protein by immunofluorescence. (G, H) Western blot and quantification of iNOS/Arg-1. Scale bars: 20 μm. Mean ± SEM; n = 3. *P* < 0.05, *P* < 0.01, *P* < 0.001, *P* < 0.0001.

To investigate changes in macrophage phenotype following MLB treatment, we performed double immunofluorescence staining for colonic M1 (CD86^+^/F4/80^+^) and M2 (CD206^+^/F4/80^+^) macrophages. In DSS-induced colitis mice, a large number of M1 macrophages infiltrated the colon, whereas M2 macrophages were nearly absent, indicating sustained M1 polarization (Fig. 7A–C). Notably, MLB administration led to a marked reduction in M1 macrophage levels and a concurrent rise in M2 macrophage presence relative to controls (Fig. 7A–C). This shift in macrophage polarization was further corroborated by qRT-PCR, which indicated downregulated expression of M1-associated genes (CD86, iNOS) and enhanced transcription of M2-associated markers (CD206, Arg-1) in colon tissue (Fig. 7B–D).

Previous findings indicate that MLB's therapeutic effects are mainly driven by its promotion of SBAs production. To determine whether SBAs contribute to macrophage phenotype regulation, we treated RAW264.7 macrophages in vitro with a mixture of four representative SBAs—DCA, LCA, HDCA, and 12-Ketolithocholic acid (12-ketoLCA)—which were significantly elevated in MLB-treated mice and present at relatively high abundance (Fig. 6G and Fig. S4E, Supporting Information). Treatment with the SBA mixture effectively suppressed LPS-induced M1 polarization, as evidenced by reduced mRNA levels of IL6, TNF-α, CD86, and iNOS alongside increased expression of the M2 marker CD206 (Fig. 7E). These findings were confirmed by immunofluorescence and Western blot analyses, which revealed reduced iNOS levels and restored Arg-1 expression after treatment with the SBA mixture (Fig. 7F–H). Overall, these results suggest that MLB mitigates colitis by increasing secondary bile acid levels, inhibiting M1 macrophage polarization, and enhancing M2 macrophage polarization in the colon.

### 4.8 Therapeutic effect of MLB in delayed treatment of colitis

**Fig. 8:**
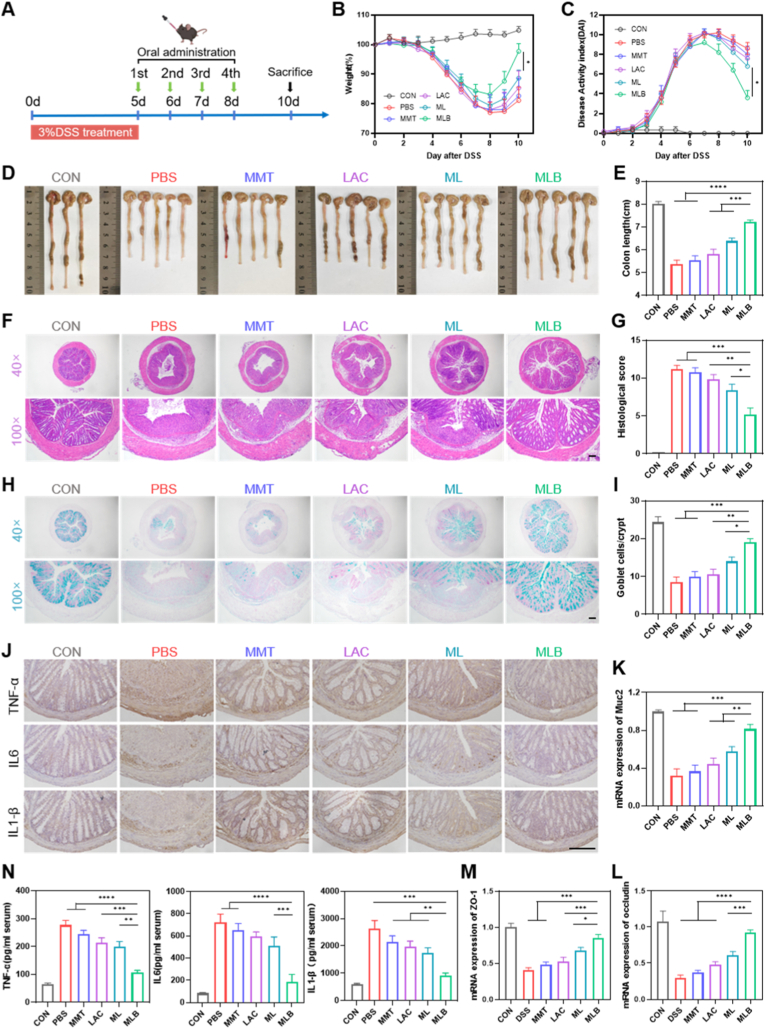
**Therapeutic effect of MLB in delayed treatment of colitis.** (A) Experimental design: C57BL/6 mice received 3% DSS in their drinking water from day 0 through day 5. From day 5 onward, mice were orally gavaged with PBS, MMT, LAC, ML, or MLB (1 × 10^8^CFU) on days 5–8. (B–E) Body weight changes, DAI, representative colon images, and colon length measurements. (F, G) H&E-stained colon sections and histopathological scores. (H, I) Alcian blue staining of colonic sections was performed, followed by counting goblet cells per crypt. (J) Immunohistochemical staining of pro-inflammatory cytokines. (K, M) Relative mRNA expression. (N) Serum concentrations of pro-inflammatory cytokines. Scale bars, 100 μm. Data are shown as means ± SEM (n = 5). ∗*P*< 0.05, ∗∗*P*< 0.01, ∗∗∗*P*< 0.001, ∗∗∗∗*P* < 0.0001. (For interpretation of the references to color in this figure legend, the reader is referred to the Web version of this article.)

To assess MLB's therapeutic potential, acute colitis was established in C57BL/6 mice by providing 3% DSS in drinking water for five days. Starting from day 5, mice received oral gavage of PBS, MMT, LAC, ML, or MLB (1 × 10^8^ CFU/mouse) on days 5, 6, 7, and 8 (Fig. 8A). Notably, MLB treatment significantly protected mice from DSS-induced acute colitis, as evidenced by reduced body weight loss, lower DAI scores, longer colon lengths, alleviated histological damage, improved colonic barrier integrity, and decreased inflammatory responses (Fig. 8B–N). These findings support the conclusion that MLB effectively mitigates DSS-induced acute colitis.

## 5 Discussion

Accumulating evidence indicates that patients with UC often exhibit gut microbiota dysbiosis, but whether this imbalance is causal remains unclear due to the limitations of observational studies [49,50]. To establish a causal link, we employed MR analysis, which reduces confounding biases inherent in observational studies [35]. Our MR analysis identified *Lactobacillus* as a genus with a genetically predicted protective effect against UC, providing a compelling rationale for targeting this bacterium therapeutically [51].

However, the clinical efficacy of *Lactobacillus* supplementation has been inconsistent, largely due to poor gastrointestinal survival and colonization [12,18,19,52]. As demonstrated by Zmora et al., host colonization resistance often limits probiotic engraftment [20]. While advanced delivery systems like microencapsulation have been developed, their complexity and safety concerns hinder clinical translation [13,16,17]. A more promising strategy involves leveraging biofilm formation, which naturally enhances bacterial resilience and persistence [21,26]. We hypothesized that MMT, a safe, mucoadhesive antidiarrheal agent, could serve as an ideal scaffold to promote probiotic biofilm formation [26].

Building on this concept, we developed the MLB platform by inducing spontaneous biofilm formation of *L. acidophilus* on MMT. MLB markedly improved bacterial survival under gastrointestinal stress, enhanced mucosal adhesion in UC mice and patient tissues, and promoted stable colonization in vivo. Importantly, the MLB biofilm can be produced through a simple and scalable co-culture process using clinically approved and readily available materials, without complex chemical synthesis. Moreover, both montmorillonite and Lactobacillus have a long history of safe human use, and our in vivo experiments did not reveal detectable adverse effects on major organ function or tissue histology. Notably, biofilm formation is achieved through a purely physical process without chemical modification, thereby minimizing potential safety concerns associated with synthetic linkers or polymers.

The therapeutic efficacy of MLB is underpinned by a sequential mechanism that bridges microbial remodeling to immune resolution [48,53]. MLB administration first restored gut microbiota homeostasis in mice with colitis, reversing the characteristic decline in diversity and *Firmicutes*, with a notable increase in *Lactobacillus*, while suppressing *Proteobacteria* expansion. This microbiota remodeling, validated by FMT to be causal, subsequently rectified the impaired microbial bile acid metabolism [44]. Specifically, MLB enhanced BSH activity, a process likely facilitated by the enriched *Lactobacillus*, leading to increased deconjugation of bile acids and a marked elevation in anti-inflammatory SBAs, a process whose critical role was confirmed by BSH inhibition [41]. Ultimately, these MLB-induced SBAs directly modulated the colonic immune milieu, driving a shift in macrophage polarization from a pro-inflammatory M1 to an anti-inflammatory M2 phenotype, thereby alleviating inflammation [54,55].

There are several limitations in our study. First, although *Lactobacillus acidophilus* was selected as a representative model strain, functional heterogeneity exists among lactic acid bacterial strains, even within the same species, which warrants caution when extrapolating our findings. Notably, the montmorillonite-enhanced biofilm strategy developed in this work is primarily driven by physicochemical interactions between montmorillonite and bacterial surface charges, rather than strain-specific biological traits. Therefore, this platform is likely applicable to other biofilm-forming lactic acid bacteria, including various *Lactobacillus* species, and may even extend to other genera such as *Bifidobacterium*, which merits further investigation. Second, although SEM and ConA staining confirmed MLB biofilm formation, we did not quantify biofilm components (e.g., EPS/proteins) or parameters such as thickness and density. This is because our study mainly focused on the biofilm's protective function in maintaining probiotic viability, which is essential for its therapeutic efficacy. Third, the integrity and colonization persistence of the MLB biofilm were not systematically evaluated under more physiologically relevant conditions, such as gut microbiota competition, regional differences along the intestinal tract, and variations in mucus layer thickness. Finally, the clinical adhesion experiments involved a limited sample size and lacked stratified analyses by clinical characteristics, which may restrict the generalizability of our findings. Larger independent studies are required to confirm these observations.

## 6 Conclusion

In conclusion, our study developed an orally administered probiotic system that improves bacterial survival and colonization throughout the gastrointestinal tract. The MLB system effectively reshapes gut microbiota composition and enhances fecal BSH activity, thereby restoring microbial bile acid metabolism. Elevated fecal SBAs promote anti-inflammatory polarization of intestinal macrophages, contributing to the resolution of inflammation. Moreover, MLB exhibits a favorable safety profile and sustained therapeutic efficacy. Collectively, this study confirms the critical role of microbiota–bile acid axis modulation in UC pathogenesis and highlights MLB as a promising microbiota-targeted therapeutic strategy.

## Funding

This work was supported by The National 10.13039/501100001809Natural Science Foundation of China [81970469, 82100591]

## CRediT authorship contribution statement

**Zhongyuan Wang:** Conceptualization, Data curation, Formal analysis, Investigation, Methodology, Project administration, Software, Supervision, Validation, Visualization, Writing – original draft, Writing – review & editing. **Feng Zhu:** Data curation, Funding acquisition, Project administration, Resources, Writing – review & editing. **Jie Zhang:** Investigation, Methodology. **Kangkang Feng:** Investigation, Methodology. **Waresi Abudourexiti:** Project administration, Resources. **Song Li:** Data curation, Methodology. **Yanzhe Guo:** Resources, Software. **Mingfei Chen:** Supervision, Validation. **Zeqian Yu:** Validation, Visualization. **Lei Zhao:** Software, Visualization. **Zhen Guo:** Project administration, Resources, Writing – review & editing. **Chao Ding:** Conceptualization, Project administration, Resources, Writing – review & editing. **Jianfeng Gong:** Conceptualization, Data curation, Funding acquisition, Project administration, Resources, Validation, Visualization, Writing – review & editing.

## Declaration of competing interest

The authors declare no conflicts of interest.

## Data Availability

GWAS summary data for gut microbiota were sourced from MiBioGen (https://mibiogen.gcc.rugnl/), and UC GWAS data from IEU OpenGWAS (https://gwas.mrcieu.ac.uk/). Sequencing data generated in this study are available in Figshare (https://doi.org/10.6084/m9.figshare.29232554). Further data can be obtained from the authors upon reasonable request.
