# Supplementary material for: Toward clinical translation: montmorillonite-enhanced Lactobacillus biofilm alleviates colitis by modulating the gut microbiota–bile acid axis
Source: Mater Today Bio. 2026 May 8;38:103211. doi: 10.1016/j.mtbio.2026.103211 (PMC13194550; doi:10.1016/j.mtbio.2026.103211)
Supplement: Multimedia component 1 [file mmc1.docx]

**Supplementary Information**

**Toward clinical translation: montmorillonite-enhanced Lactobacillus biofilm alleviates colitis by modulating the gut microbiota–bile acid axis**


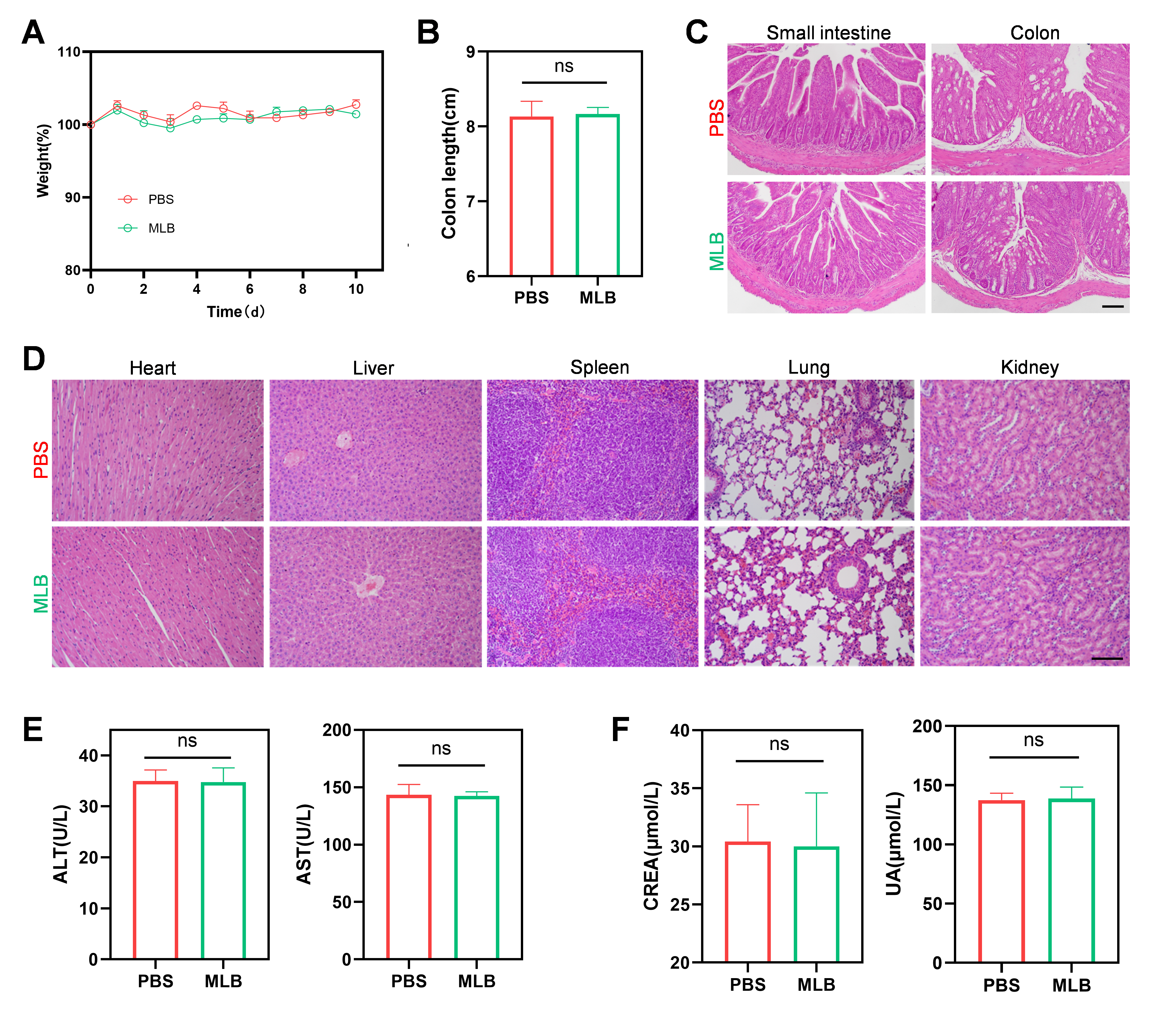


**Figure S1.** Biosafety evaluations of MLB in mice. [A, B] Weight changes during administration and colon length measured after dissection. C, D] H&E staining images of representative organs and tissues. [E, F] Liver and kidney function markers of mice after different treatments. Scale bars, 100 μm. Data are presented as means ± SEM [n = 5]. ns: non-significance.


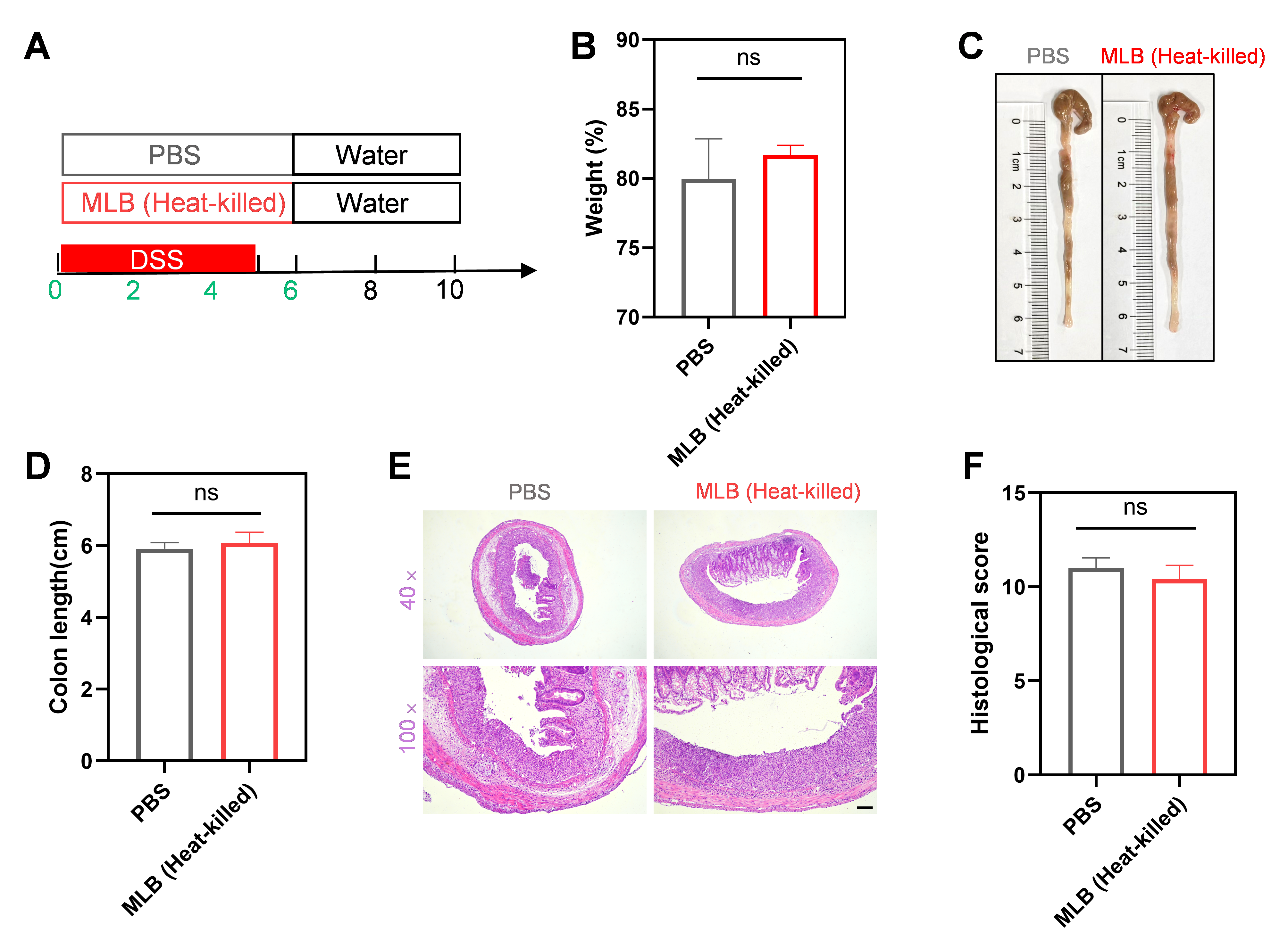


**Figure S2.** Heat-inactivated MLB loses its therapeutic efficacy against colitis. [A, B] Study design and body weight change of mice after different treatments with FMT. [C, F] Representative images of mouse colons, colon length measurements, colonic H&E staining, and histological damage scoring after different treatments. Scale bars, 100 μm. Data are presented as means ± SEM [n = 5]. ns: non-significance.


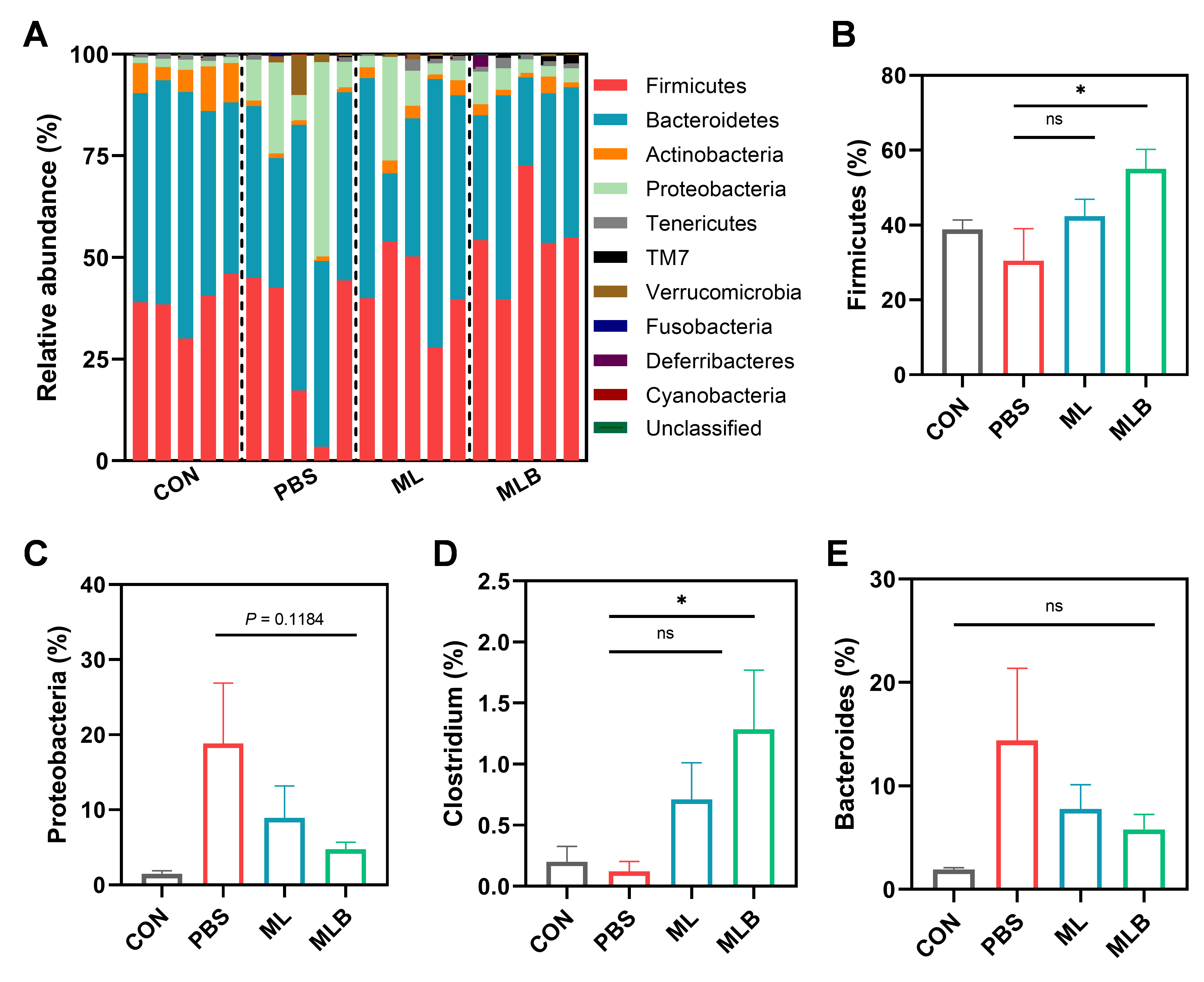
**Figure S3.** MLB modulates gut microbiota during colitis treatment. [A] Relative abundance of Lactobacillus at phylum level. [B-E] Relative abundance of Firmicutes, Proteobacteria, Clostridium and Bacteroides. Data are presented as means ± SEM [n = 5]. **P* < 0.05, ***P* < 0.01, ****P* < 0.001, *****P* < 0.0001, ns: non-significance.


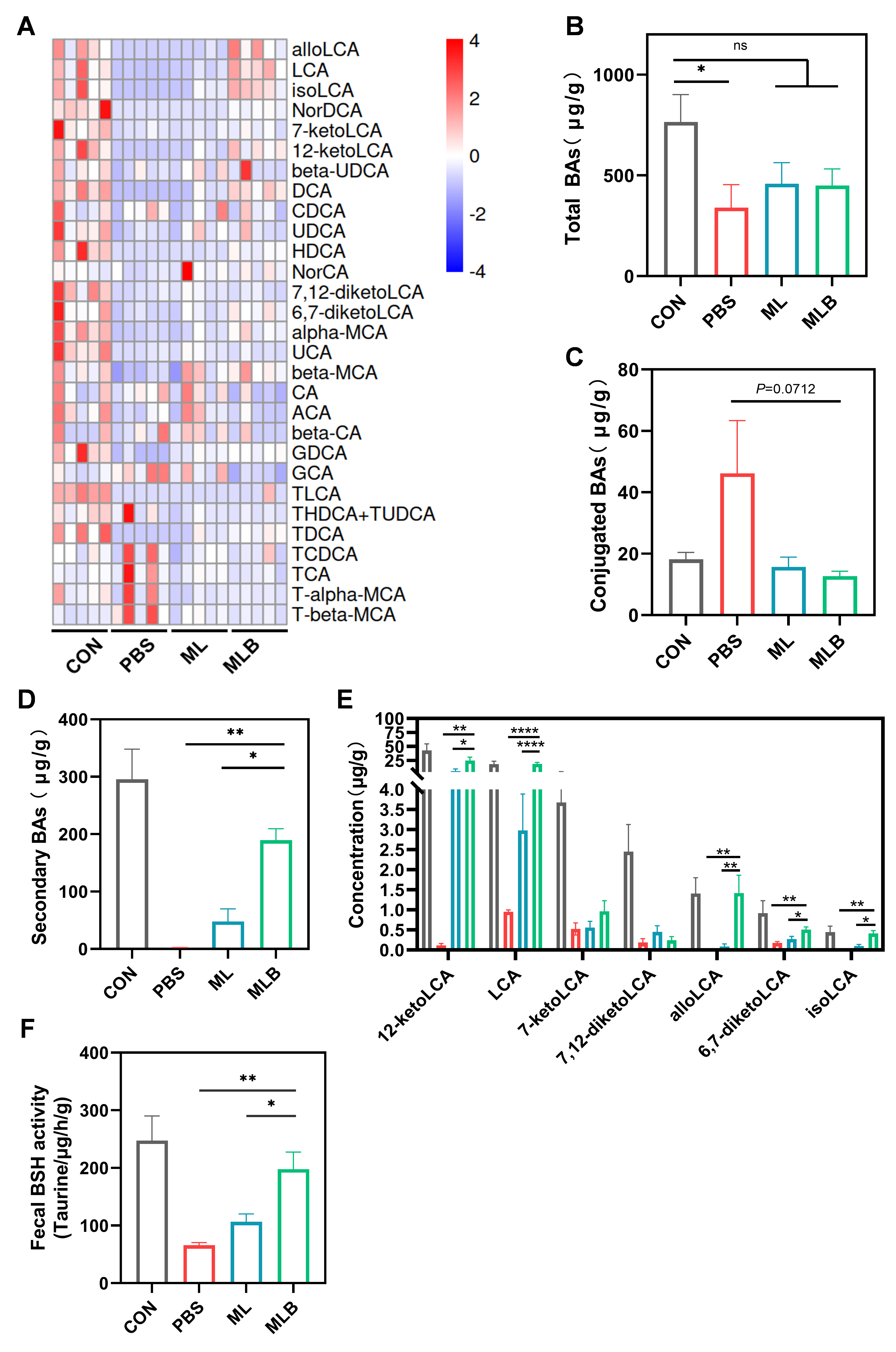


**Figure S4.** MLB alleviates colitis by modulating bile acid metabolism. [A] Heatmap showing fecal bile acid profiles across different treatment groups. [B-D] Concentrations of total, conjugated, and secondary bile acids in fecal samples from different treatment groups. [E] Concentration of LCA and their derivatives in fecal samples from different treatment groups. Data are presented as means ± SEM [n = 5]. **P* < 0.05, ***P* < 0.01, ****P* < 0.001, *****P* < 0.0001, ns: non-significance.


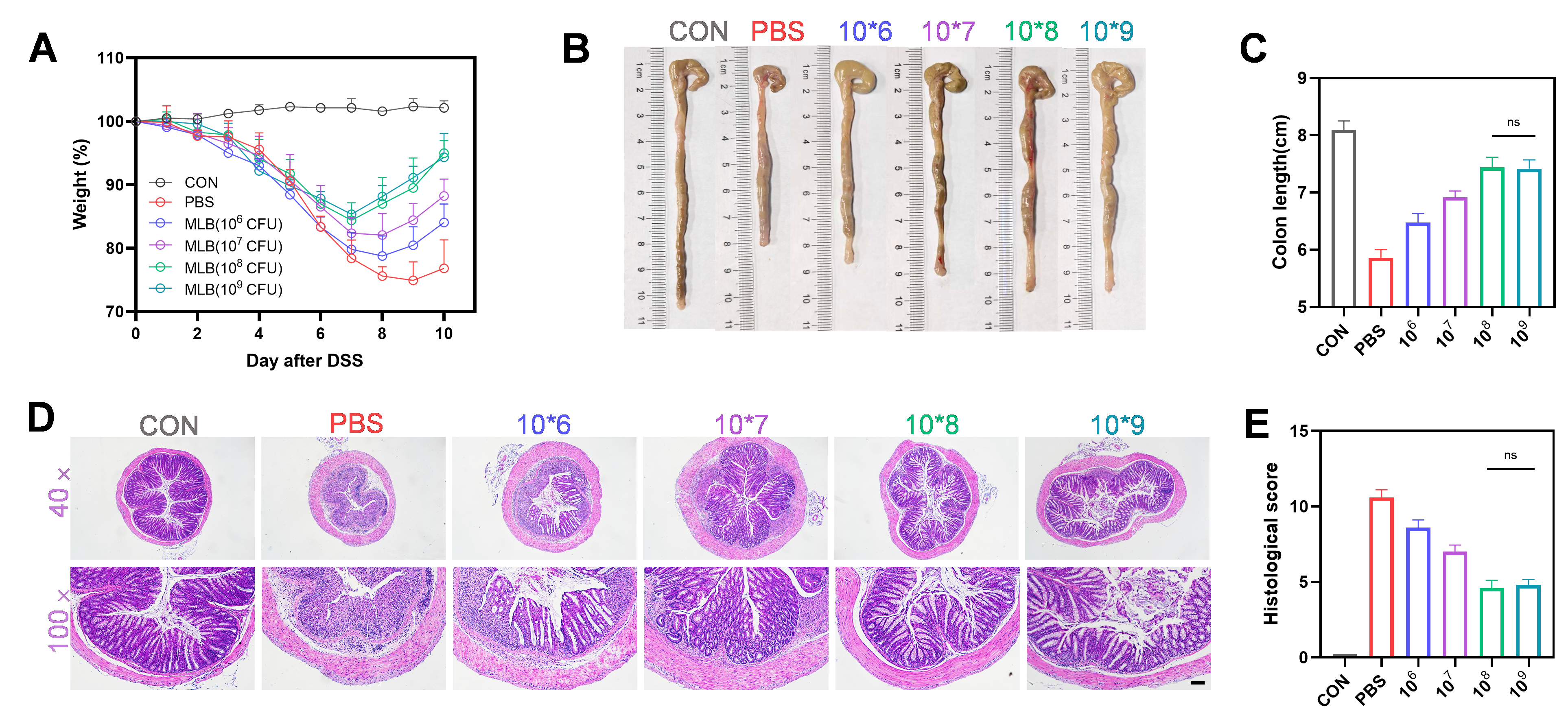


**Figure S5.** A dose escalation study of MLB in DSS-induced colitis. [A] Body weight changes during treatment. [B-C] Colon morphology and length. [D-E] H&E staining and histological scores. Scale bars, 100 μm. Data are presented as means ± SEM [n = 5]. ns: non-significance.


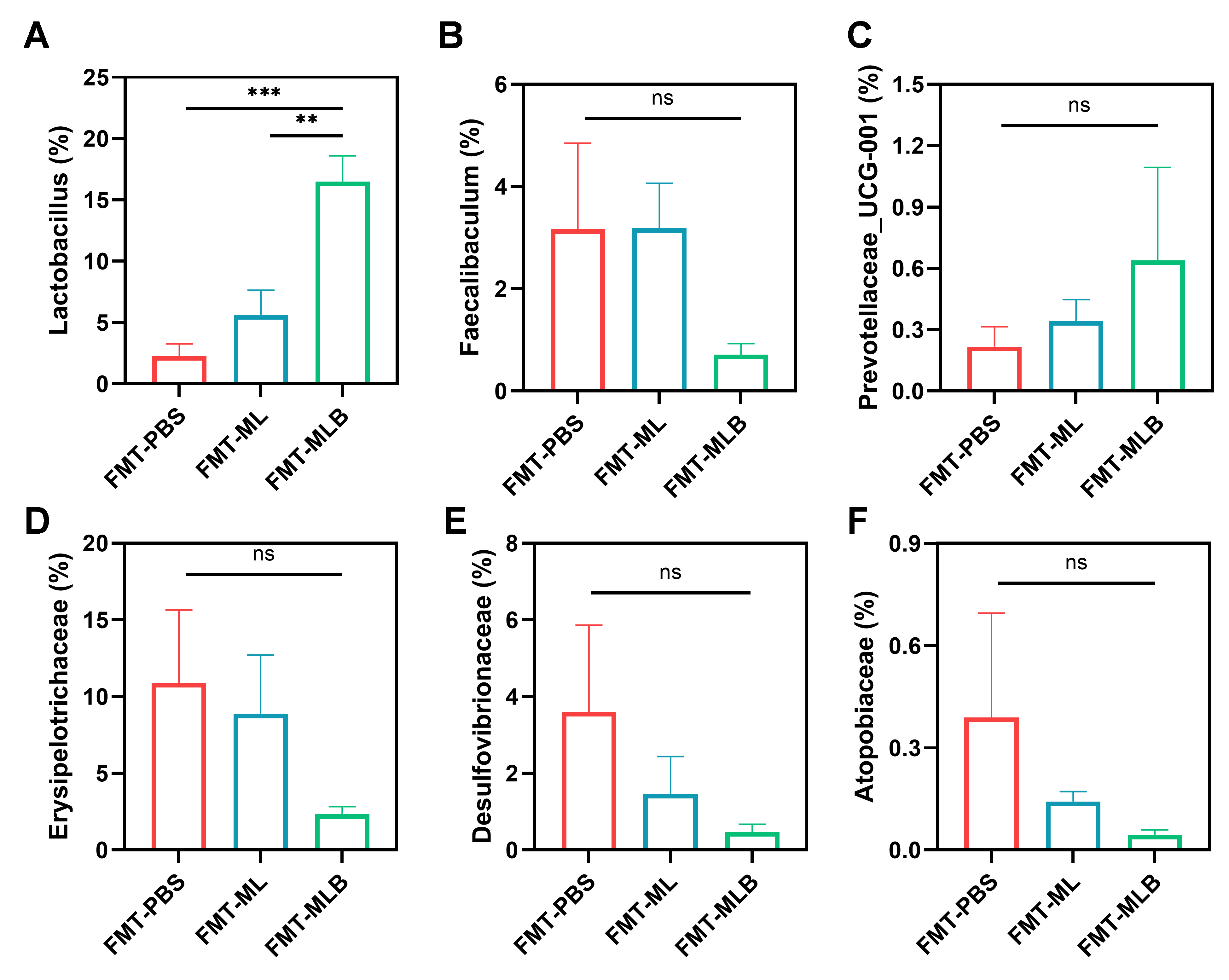


**Figure S6.** Relative abundances of representative bacterial taxa in recipient mice after FMT from different donor groups. (A–F) Relative abundances of (A) *Lactobacillus*, (B) *Faecalibaculum*, (C) *Prevotellaceae_UCG-001*, (D) *Erysipelotrichaceae*, (E) *Desulfovibrionaceae*, and (F) *Atopobiaceae*. Data are presented as means ± SEM [n = 5]. **P* < 0.05, ***P* < 0.01, ****P* < 0.001, *****P* < 0.0001, ns: non-significance.


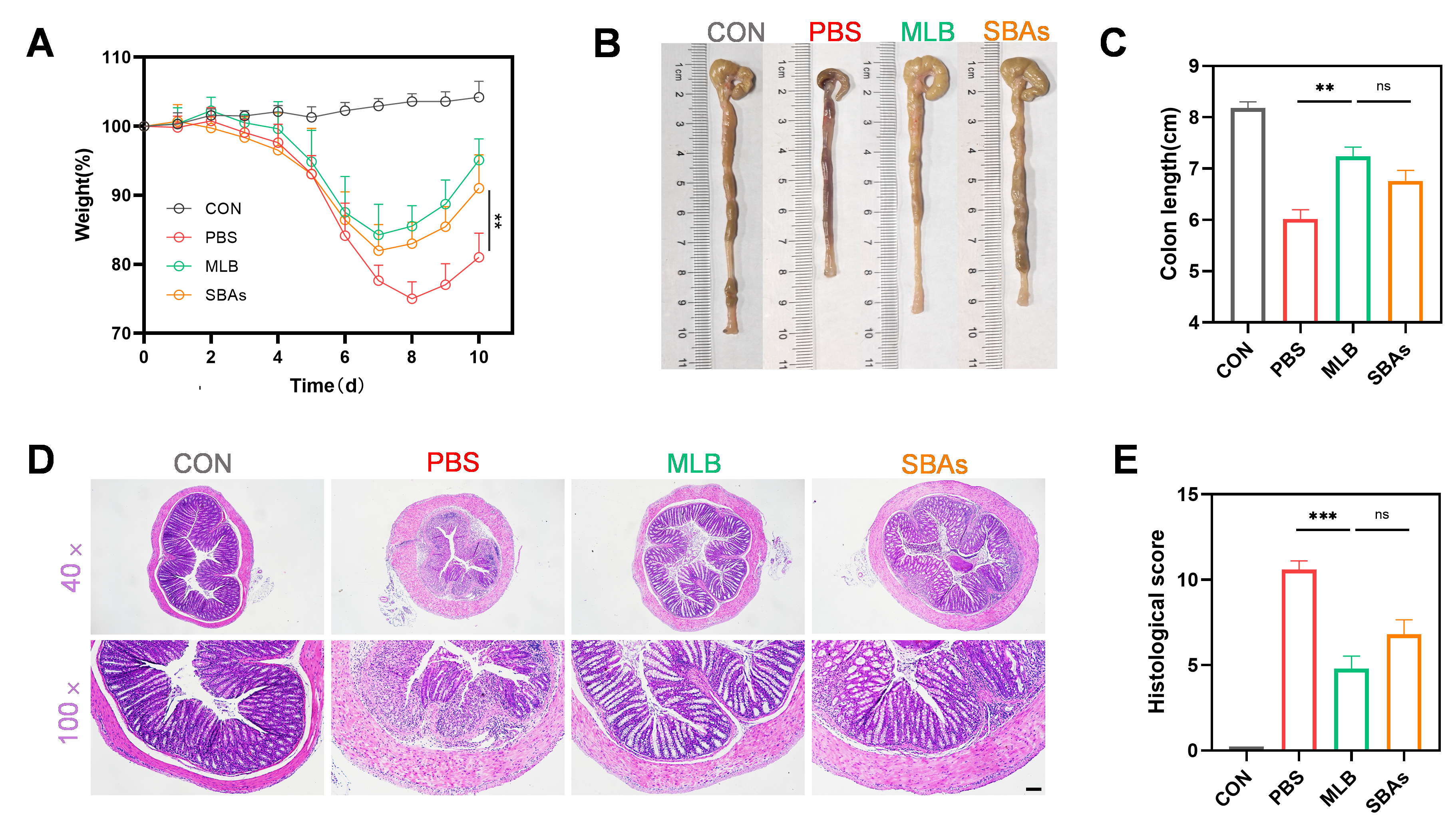


**Figure S7.** MLB alleviates colitis by modulating bile acid metabolism. [A] Body weight changes during treatment. [B-C] Colon morphology and length. [D-E] H&E staining and histological scores. Scale bars, 100 μm. Data are presented as means ± SEM [n = 5]. **P* < 0.05, ***P* < 0.01, ****P* < 0.001, *****P* < 0.0001, ns: non-significance.

**Table S1.** Primers used in this study

| **Primers** | **Forward Primer** | **Reversed Primer** |
| --- | --- | --- |
| 16srRNA | 5'-AGAGTTTGATCMTGGCTCAG-3' | 5'-TACGGYTACCTTGTTACGACTT-3' |
| LuxS | 5'-ATGACCAATCGTCAGTGGG-3' | 5'-TTAAGGCTTTCGTTGCGTG-3' |
| Tuf | 5'-ATGGTGACAAGGAAATTATGG-3' | 5'-TCATCATCACCGTTAGCAG -3' |
| Fba | 5'-ATGGTTGTTGAAGACAGTTCG -3' | 5'-TCACCTGTTGTAGAGTAAACG-3' |
| Gap | 5'-ATGTCGGAATCGACGATTGC-3' | 5'-TTATCACCGTATCCAGGACG-3' |
| β-actin | 5'-GTGAAAAGATGACCCAGATCAT-3' | 5'-GCTTCTCTTTGATGTCACGCACGAT-3' |
| IL-6 | 5'-CTGGTCTTCTGGAGTACCATAGC-3' | 5'-CTGAAGGACTCTGGCTTTGTC-3' |
| IL-1β | 5'-TCCTTCATCTTTGAAGAAGAGCCC-3' | 5'-ATGGGAACGTCACACACCAG-3' |
| TNF-α | 5'-TCTTCTCATTCCTGCTTGTGG-3' | 5'-GAGGCCATTTGGGAACTTCT-3' |
| IL-10 | 5'-ACCTGGTAGAAGTGATGCCC-3' | 5'-AGAAATCGATGACAGCGCC-3' |
| ZO-1 | 5'-GAGCGGGCTACCTTACTGAAC-3' | 5'-GTCATCTCTTTCCGAGGCATTAG-3' |
| Occludin | 5'- TTGAAAGTCCACCTCCTTACAGA-3' | 5'-CCGGATAAAAAGAGTACGCTGG-3' |
| MUC-2 | 5'- TCCTGACCAAGAGCGAACAC-3' | 5'-ACAGCACGACAGTCTTCAGG-3' |
| CD86 | 5'-TCAATGGGACTGCATATCTGCC-3' | 5'-GCCAAAATACTACCAGCTCACT-3' |
| CD206 | 5'-CAAGGAAGGTTGGCATTT-3' | 5'-CCTTTCAGTCCTTTGCAAGC-3' |
| iNOS | 5'-GCTCGCTTTGCCACGGACGA-3' | 5'-AAGGCAGCGGGCACATGCAA-3' |
| Arg-1 | 5'-CTATGTGTCATTTGGGTGGA-3' | 5'-TCTGGGAACTTTCCTTTCAG-3' |
